# The Significance of Diffusion-Weighted Imaging and Apparent Diffusion Coefficient in the Diagnosis of Early Acute Interstitial Edematous Pancreatitis

**DOI:** 10.7759/cureus.71051

**Published:** 2024-10-08

**Authors:** Sudhir K Yadav, ling liu, Penglou Zheng, Vivek Dhakal, Sanjyoti Shah, Abhishek Adhikari, Ravi Kanodia

**Affiliations:** 1 Department of Radiology, The First Affiliated Hospital of Dali University, Dali, CHN; 2 Department of Radiology, Nepal Mediciti Hospital, Kathmandu, NPL; 3 Department of Radiology, Dali Bai Autonomous Prefecture People’s Hospital, Dali, CHN; 4 Department of Radiology, Leighton Hospital, Crewe, GBR; 5 Department of General Medicine, Nepalgunj Medical College, Nepalgunj, NPL; 6 Department of Radiation Oncology, All India Institute of Medical Sciences, New Delhi, IND

**Keywords:** acute interstitial edematous pancreatitis, apparent diffusion coefficient (adc), daignosis, diffusion-weighted imaging (dwi), early detection, early diagnosis

## Abstract

The study assessed the diagnostic value of diffusion-weighted imaging (DWI) and mean apparent diffusion coefficient (ADC) in early acute interstitial edematous pancreatitis (AIEP). Fifty-six AIEP patients and 60 healthy controls underwent an upper abdominal magnetic resonance (MR) examination. The pancreas diameter and ADC values were measured and analyzed across age and gender groups. Results showed that the pancreas diameter varied with sex and age, with smaller diameters in females and the elderly. Mean ADC values were not significantly affected by age or sex. Signal changes were observed in AIEP patients' images, with DWI showing a higher positive rate than T1WI and T2WI. Comparing the control and AIEP groups, the pancreas diameter was significantly larger in AIEP patients, particularly the pancreatic tail in males. ADC values were significantly lower in AIEP patients, and the difference was more pronounced at b=800 s/mm² compared to b=500 s/mm². Mean ADC exhibited good diagnostic potential for early AIEP, with higher sensitivity and specificity values than serum amylase levels. The area under the receiver operating characteristic (ROC) curve (AUC) indicated that the diagnostic value was better at b=800 s/mm² than at b=500 s/mm².

In conclusion, the study found that normal pancreas diameter is influenced by sex and age, with smaller diameters in females and the elderly. Mean ADC values were not affected by these factors. Early AIEP could not be diagnosed solely based on pancreas diameter changes. DWI combined with Mean ADC demonstrated high diagnostic value for early AIEP.

## Introduction

Inflammation of the pancreas is caused by malfunctioning digestive enzymes in the pancreas resulting in autolysis of acinar cells and the development of edema in acute pancreatitis. This pathological process can trigger systemic inflammatory response syndrome (SIRS), which may cause multiple organ failure and other life-threatening complications [1]. In the past few decades, acute pancreatitis has increased, particularly among 50-70-year-old middle-aged individuals, with a higher prevalence among males. The mortality rate tends to rise with increasing age, mainly after 70 years. It is remarkable that while the incidence has increased, the United States has witnessed a decrease in mortality, with recent studies reporting a mortality rate of approximately 2% [2]. The dominant etiology for acute pancreatitis is alcohol and gallstones [3].

The revised Atlanta classification categorizes acute pancreatitis into the following two main types: interstitial edematous pancreatitis, which constitutes 80-90% of cases and is characterized by diffuse localized enlargement of the pancreatic parenchyma with heterogeneous or occasionally homogeneous enhancement and peripancreatic fat stranding or fluid collection. This type of pancreatitis mostly resolves within one week. Necrotizing pancreatitis is another type of pancreatitis that affects the pancreas and peripancreatic tissue. In acute pancreatitis, pancreatic tissue involvement is more common as compared to peripancreatic tissue changes. The severity of pancreatitis is categorized into the following three levels: mild acute pancreatitis (no systemic or local complications), moderate acute pancreatitis (lasting ≤48 h and/or associated with local or systemic complications), and severe acute pancreatitis (persistent organ failure lasting ≥48 h). There are the following two phases to characterize acute pancreatitis: the early phase, characterized by a cytokine cascade leading to SIRS, usually occurring within the first week and lasting for up to two weeks, and the late phase, which occurs in patients with moderate-to-severe pancreatitis and is characterized by persistent systemic signs of inflammation or the development of systemic or local complications [4]. For acute pancreatitis diagnostic criteria established by the International Association of Pancreatology require the presence of at least two out of the following three criteria: (a) elevated laboratory findings with serum amylase or lipase levels exceeding three times the upper limit of the normal range, (b) upper abdominal pain, and (c) compatible imaging findings using techniques such as CT, MRI or ultrasound [5]. Assessments and diagnoses of acute pancreatitis are performed by various imaging modalities, with the choice of modality determined by the clinical history and severity and of the disease. In mild cases, imaging may not be necessary for patient management, except for identifying the underlying cause of acute pancreatitis.

Computed tomography (CT) is a readily available imaging technique that allows for the prompt acquisition of images. This is particularly valuable in cases of critically ill patients. However, a significant associated risk is the potential for contrast-induced nephrotoxicity, especially in patients with deranged renal function. In the early stages of acute pancreatitis, the accurate diagnosis of pancreatic necrosis can be challenging. Pancreatic parenchymal perfusion appears diffusely or focally heterogeneous due to various degrees of edema, which can be misinterpreted as pancreatic necrosis [6]. These findings are often observed in poorly enhancing areas of acute pancreatitis and have an accuracy of less than 30% [7].

To assess the severity and clinical prognosis of acute pancreatitis, the CT severity index (CTSI) was introduced by Balthazar et al. in 1990. CTSI evaluates acute pancreatitis based on pancreatic parenchymal changes and the presence of pathological fluid accumulations [7]. Radiologists are tasked with determining the presence and extent of necrosis, which is semi-quantified as less than 30%, 30-50%, or more than 50% [8]. A modified version, known as m-CTSI, was introduced in 2004 by Mortele et al., which simplifies the diagnosis of pancreas and peripancreatic inflammation extent. However, CTSI has limitations in predicting acute pancreatitis in association with extrapancreatic manifestations. Despite this, both CTSI and m-CTSI are equivalent to clinical Acute Physiology and Chronic Health Evaluation II (APACHE II) scores in diagnosing acute pancreatitis severity and predicting the need for intervention [9].

In mild acute interstitial edematous pancreatitis, the accuracy of CT diagnosis is limited, with approximately one-third of patients revealing similar findings to those with a normal pancreas [9]. The classical CT findings in this form of pancreatitis include peripancreatic inflammatory changes, such as fluid and fat stranding. These inflammatory changes in the pancreatic parenchyma appear homogeneously enhanced in contrast studies. In contrast, the loss of enhancement in necrotizing acute pancreatitis differentiates it from acute interstitial edematous pancreatitis. Clinical scoring indices are more specific than CT for predicting severity at the time of admission, as there is a consistent correlation between clinical and laboratory data. Confirmatory diagnosis in these cases may require a follow-up CT scan, as the findings can be indeterminate in the early stages. Pancreatic necrosis cannot be accurately diagnosed within 72 hours of the onset of acute pancreatitis, with the most accurate diagnosis occurring between five and seven days after the onset. This is why follow-up and initial contrast-enhanced CT scans are recommended for high-risk patients to minimize adverse complications [10].

Magnetic resonance imaging (MRI) has seen significant advancements in recent years, with the introduction of multi-channel phased array coils, parallel imaging, respiratory-triggered techniques, and navigator echo-based methods. These improvements make it easier to obtain faster images and motion-resistance and have enhanced spatial resolution. MRI is particularly useful for diagnosing acute pancreatitis, surpassing CT in terms of diagnostic outcomes. Recently, the magnetic resonance severity index (MRSI) has been proposed for the diagnosis of acute pancreatitis, and it correlates significantly with Ranson's scores, CTSI, C-reactive protein (CRP) levels, hospital admission intervals, and clinical prognosis. Additionally, MR cholangiopancreatography (MRCP) sequences allow for the non-invasive assessment of incidental findings, such as cholelithiasis, pancreatic duct anatomy variations (e.g., underlying pancreas divisum), and duct integrity (disconnected pancreatic duct syndrome) [11].

Specific MRI sequences, such as fat-saturated T1-weighted and out-phase sequences, can provide a clear view of the normal anatomy, size, shape, configuration, and attenuation of the pancreas. In acute pancreatitis, the pancreatic areas appear isointense to hypointense on T1-weighted images and isointense to mildly hyperintense on T2-weighted images. Peripancreatic edema, fat stranding, and fluid stranding are well-visualized on fat-saturated T2-weighted sequences. Focal or diffuse inflammatory changes in the pancreas parenchyma associated with acute pancreatitis typically show homogenous enhancement during gadolinium contrast on T1-weighted images. In contrast, necrotic pancreatic parenchyma exhibits heterogeneous intensity with no enhancement. MRI provides significant advantages over CT for the early diagnosis of internal constituents and the characterization of collections and walled-off pancreatic necrosis compared to simple pseudocysts [12].

Diffusion-weighted MRI (DWI) is a relatively new addition to the prospect of diagnostic tools and plays a vital role in characterizing both focal and diffuse lesions of parenchymal disease in abdominal imaging. DWI relies on the theory of Brownian motion of water protons within living biological tissues, which is influenced by cellularity and cell membrane status. Tissues with pathologically disrupted membranes exhibit greater water molecule movement. In particular, conditions such as cytotoxic edema, abscesses, and tumors can demonstrate a restricted diffusion pattern on DWI. The sensitivity of DWI sequences can be adjusted by varying the b coefficient, which represents the strength of the diffusion sensitizing gradient and is measured in seconds per square millimeter (s/mm^2^). The apparent diffusion coefficient (ADC) is a quantitative parameter that reflects the combined effects of water diffusion in the extracellular space and perfusion in the capillary network. ADC values are derived from the post-processing of DWI and presented as parametric maps, with different ADC values for various tissues. Malignant lesions, which typically have higher cellularity associated with decreased diffusion, appear hyperintense on high b-value DWI images with corresponding low ADC values compared to adjacent normal parenchyma. While DWI is sensitive to malignant tumors, it has also shown promise in characterizing pancreatic masses. There are a minimum of four b values: 0, 150, 400-500, and 800-1,000 s/mm^2^ in DWI sequences for the typical involvement of the pancreas in the inflammatory phase [13,14].

In conclusion, acute pancreatitis is a complex and potentially life-threatening condition that requires assessment of accurate diagnosis and severity. Various imaging modalities, such as CT, and MRI (DWI), play pivotal roles in this process. While CT remains readily available and suitable for critically ill patients, MRI, with its evolving techniques and greater spatial resolution, offers advantages in diagnosing and characterizing acute pancreatitis. DWI has emerged as a valuable tool for characterizing abdominal lesions, including pancreatic masses, and shows promise in the evaluation of acute pancreatitis. Assessment of severity and accurate diagnosis are essential for guiding appropriate treatment strategies in acute pancreatitis.

## Materials and methods

In this study, we investigated the diagnostic potential of magnetic resonance imaging (MRI) in early acute interstitial edematous pancreatitis (AIEP). A total of 56 patients were included in this research, from the First Affiliated Hospital of Dali University, spanning from January 2017 to March 2018. These patients comprised 26 females and 30 males, categorized into different subgroups based on sex and age. Moreover, 60 normal subjects (30 for each sex) were selected for comparison, considering age subgroups. All subjects underwent abdominal MRI scans meeting specific quality and diagnostic criteria.

Inclusion and exclusion criteria

Patients included in the study met specific criteria. Inclusion criteria included individuals experiencing sudden, acute, persistent, and severe upper abdominal pain. Their serum amylase activity exceeded three times the upper limit of the normal range. Exclusion criteria involved patients with previous confirmation of acute pancreatitis through CT or MRI, those with upper abdominal pain lasting over a week, and those with poor cooperation or poor image quality.

Clinical data of subjects

The AIEP group's age ranged from 19 to 72 years, with a median age of 45.0 years. Out of the 56 AIEP patients, 35 showed a significant increase in serum amylase levels. The control group consisted of individuals aged between 18 and 75 years, with a median age of 43.5 years, where 37 patients had undetectable serum amylase levels.

Equipment and methods

The MRI scanning equipment used was a 1.5T-HDe superconducting magnetic resonance scanner with an eight-channel phased array coil. Before scanning, patients underwent fasting for at least 8 h and received breathing training while in a supine position. The MRI scan sequences and parameters used were specific to the purpose of the study.

Data analysis and statistical methods

The collected MRI data were analyzed by three radiologists independently, focusing on signal changes in T1WI, T2WI, and DWI sequences. The anteroposterior diameter of the pancreatic head, body, and tail was measured under the fat-suppressed T2WI sequence. The pancreatic head was measured from the anterior aspect of the inferior vena cava, the body of the pancreas was vertically connected from the left vertebral body margin to the body of the pancreas, and the tail of the pancreas was vertically connected from the inner margin of the left kidney to the tail of the pancreas. The data of DWI were processed by the Functool software package (Chicago, IL: GE Healthcare), which is provided by the equipment. Apparent diffusion coefficient (ADC) diagrams were automatically generated from inbuilt software. The mean ADC values of the normal pancreatic tissue and the focus area of acute interstitial edematous pancreatitis were measured by DWI. The size of ROI was 20-40 mm^2^. ROI was located in the center of the lesion as far as possible, avoiding the artifacts of large vessels and their branches, the main pancreatic duct, and pancreatic duct excretion. In the control group, three ROIs were measured at the head, body, and tail of the pancreas of the AIEP group. Statistical analysis was carried out using the SPSS 19.0 software (Armonk, NY: IBM Corp.) package. Measurement data were expressed as mean±standard deviation. An independent sample t-test was carried out if the normal distribution was satisfied, and if the distribution was not satisfied, the rank-sum test was used. Diagnostic experimental analysis was carried out for mean ADC value, and the optimal threshold of mean ADC (ADC value at the sum of sensitivity and specificity) was determined, and the receiver operating characteristic (ROC) curve was compared. The area of sensitivity and specificity of counted data were tested by χ^2^ test; test standard a=0.05 (i.e., when p<0.05, the difference was statistically significant).

## Results

Key findings

Pancreatic Diameter Changes

The study showed that the normal values for pancreatic diameter varied by gender and age. Male subjects had larger pancreas diameters than females, with diameters decreasing with age. Male elderly subjects exhibited smaller pancreas diameters than their younger counterparts, and males in middle age had smaller pancreatic head diameters compared to young males. The older female group had decreased diameters in all three pancreatic regions compared to the younger and middle-aged female groups. Differences were statistically significant.

Changes in Pancreatic ADC Values

**Figure 1:**
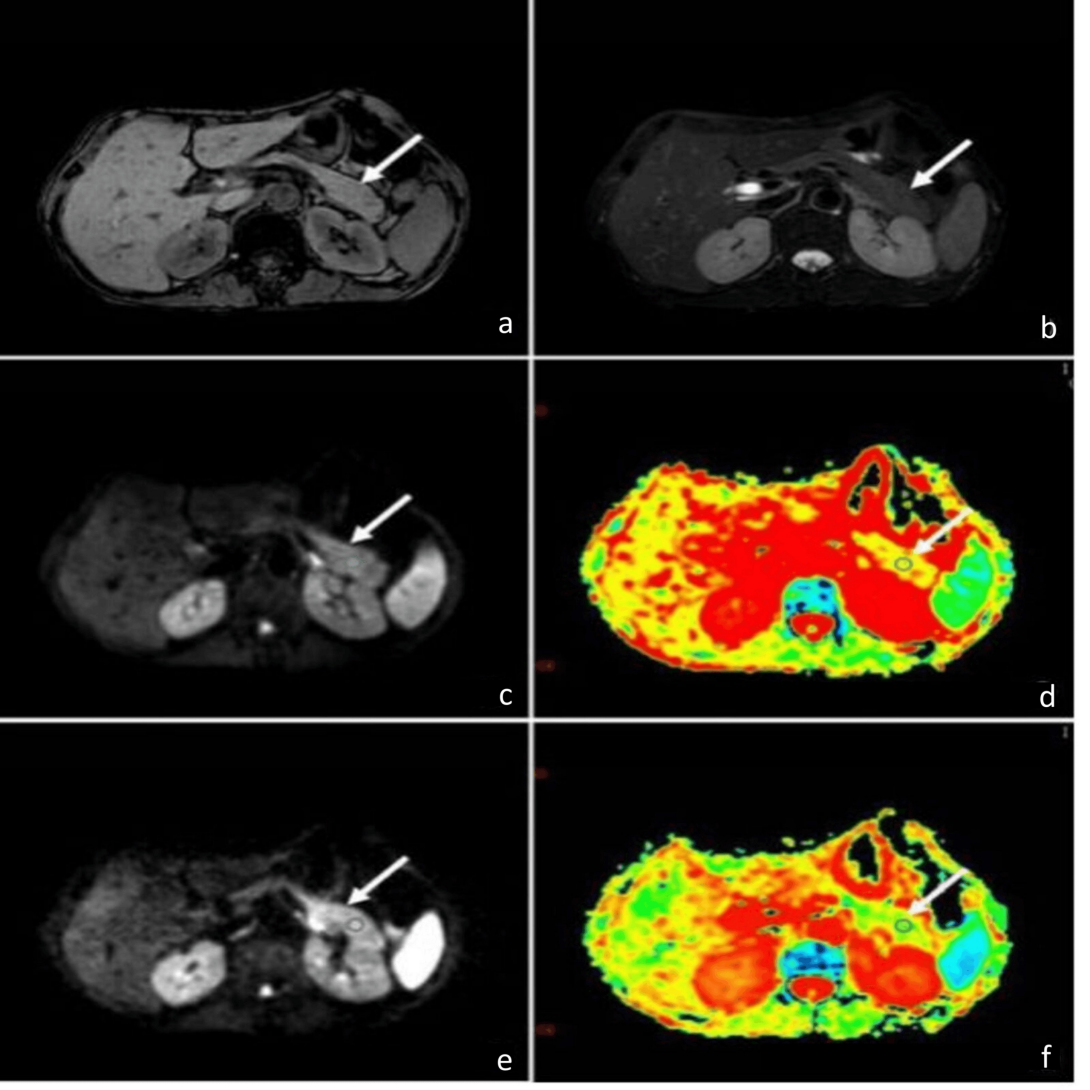
MRI findings of a 42-year-old female presenting with 14 hours of abdominal pain. (a) T1-weighted imaging (T1WI) showed the pancreas with an isointense signal, and the pancreatic tail measured 2.2 cm. (b) T2-weighted imaging (T2WI) with fat suppression also demonstrated an isointense signal throughout the pancreas. (c) Diffusion-weighted imaging (DWI) at b=500 s/mm² revealed slightly increased signal intensity in the pancreatic tail. (d) Apparent diffusion coefficient (ADC) mapping at b=500 s/mm² indicated a mean ADC of 1.53x10⁻³ s/mm², with the pancreatic tail showing a yellow-green color, suggestive of restricted diffusion. (e) DWI at b=800 s/mm² showed more pronounced high signal intensity compared to b=500 s/mm². (f) ADC mapping at b=800 s/mm² demonstrated a mean ADC of 1.41x10⁻³ s/mm², with a larger area of green indicating further restricted diffusion compared to b=500 s/mm². These findings suggest possible pancreatic pathology, correlating with the patient's elevated blood amylase levels.

The study found no significant correlation between gender and age with pancreatic ADC values but noted that ADC values decreased with increasing b values. The study observed that, with b=800 s/mm^2^, the optimal threshold for mean ADC improved diagnostic sensitivity and specificity compared to b=500 s/mm^2^ as given in Figures 1a-1f.

Comparison of Diagnostic Efficacy

Mean ADC values at b=800 s/mm^2^ were found to be more effective in diagnosing AIEP than mean ADC values at b=500 s/mm^2^.

MRI Diagnosis of Early AIEP

MRI, particularly DWI, showed significance in diagnosing early AIEP. The study emphasized the importance of using DWI and its superior sensitivity and specificity compared to traditional imaging techniques. DWI's ability to detect tissue edema early in AIEP, due to microcirculatory disturbances in the pancreas, made it a valuable diagnostic tool (Table 1).

**Table 1:** Signals changes in the region of interest of pancreatic and peripancreatic lesion.

Groups	Signals change			Peripancreatic effusion	Peripancreatic fat stranding
	T1WI	T2WI	DWI		
Normal control group (30 males and 30 females)	Isointense signal 60 cases	Isointense signal 60 cases	Isointense signal 60 cases	0 cases	0 cases
AIEP group (36 males and 20 females)	Low or slightly low signal (27/56)	High or slightly high signal (35/56)	High or slightly high signal (45/56)	33/56	15/56
χ^2 ^value	12.600	4.375	-	6.081	32.308
Comparison of positive rate of DWI	0.000	0.036	-	0.014	0.000

Value of DWI Combined With Mean ADC

**Figure 2:**
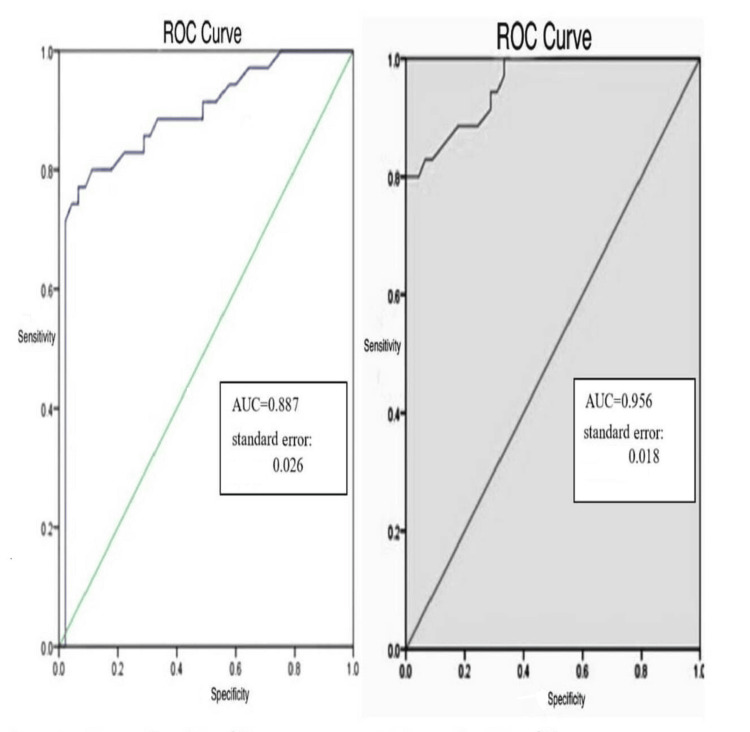
Comparison of ROC curves at different diffusion weightings (b=500 s/mm² and b=800 s/mm²). ROC: receiver operating characteristic; AUC: area under the curve

The combination of DWI and mean ADC values at specific b values enhanced the diagnostic capability for AIEP. The ROC curves demonstrate the diagnostic performance for differentiating between conditions using diffusion-weighted imaging at two b-values (500 s/mm² and 800 s/mm²). The AUC for b=500 s/mm² is 0.887 (standard error=0.026), and for b=800 s/mm², the AUC is 0.956 (standard error=0.018), indicating superior diagnostic accuracy at the higher b-value (Figure 2).

## Discussion

The key to making an accurate and timely diagnosis of AIEP lies in utilizing a mutual approach that combines clinical data with advanced imaging techniques, particularly, diffusion-weighted imaging (DWI) and apparent diffusion coefficient (ADC) values.

Significance of serum amylase (AMY) in early AIEP

While acknowledging its limitations, the authors emphasize the importance of serum amylase (AMY) levels in the early diagnosis of acute interstitial edematous pancreatitis (AIEP). Serum amylase is a common laboratory indicator for acute pancreatitis (AP) diagnosis, but its sensitivity and specificity are subject to various factors, including time, treatment interventions, and individual variations. However, the study points out that the degree of increase in serum amylase may not always correspond with the severity of the condition. The sensitivity of serum amylase in this study was found to be 62.5%, lower than the meta-analysis results, possibly due to post-admission treatment affecting amylase levels. This study highlighted the importance of combining abnormal serum amylase results with imaging examinations for a more accurate diagnosis.

The principle of DWI and the significance of b value

In this study, the principles of DWI are examined, emphasizing its ability to detect the Brownian motion of water molecules that reflect tissue characteristics. The authors explain the significance of the b value in DWI, emphasizing its influence on image quality and the apparent diffusion coefficient (ADC) value. For abdominal examinations, it's advisable to choose a higher b value, preferably in the range of 600-1000 s/mm^2^, to minimize the impact of perfusion factors and boost the accuracy of ADC value. The discussion underscores the importance of choosing an appropriate b value for a clearer diagnosis and differential diagnosis of pancreatic lesions.

MRI diagnosis of early AIEP

This section explores how magnetic resonance imaging (MRI) helps in diagnosing acute interstitial early pancreatitis (AIEP) in its early stages. The study investigates pancreas morphology, T1-weighted imaging (T1WI), T2-weighted imaging (T2WI), DWI signals, peripancreatic collection, peripancreatic fat stranding, and ADC values. Notably, the authors find gender and age-related variations in normal pancreatic diameter and ADC values. They discuss how age and gender can influence the measurements of the pancreas, stressing the importance of gender-specific and age-specific criteria to avoid misdiagnosis.

Value of DWI combined with mean ADC value in AIEP diagnosis

This study assesses the diagnostic value of DWI combined with mean ADC values for diagnosing AIEP. The results indicate that the mean ADC values in the AIEP group are significantly lower than those in the normal control group, with differences observed at different b values. The authors suggest that the combination of DWI and mean ADC values enhances diagnostic accuracy. They give sensitivity, specificity, and area under the receiver operating characteristic curve (AUC) values for various b values, highlighting that DWI with a b value of 800 s/mm^2^ outperforms other b values in terms of diagnostic performance [15].

This study reveals that relying solely on serum amylase levels for early AIEP diagnosis is limited and advocates the use of imaging techniques, particularly DWI with appropriate b values. For an accurate and timely diagnosis of AIEP, the study recommends a comprehensive approach combining clinical data and advanced imaging techniques, emphasizing the importance of gender and age-specific considerations in interpreting imaging results.

## Conclusions

In conclusion, this research emphasizes the potential of using magnetic resonance imaging, most importantly DWI sequences and mean ADC values, for the early diagnosis of acute interstitial edematous pancreatitis (AIEP). These sequences increase the sensitivity and specificity of detecting AIEP, overcoming the limitations of serum amylase. Moreover, gender and age differences of the patient play an important role in interpreting the imaging results. The main objective of this study was to evaluate the diagnostic efficacy of diffusion-weighted imaging (DWI) and the mean apparent diffusion coefficient (ADC) in the early detection of acute interstitial edematous pancreatitis (AIEP), intending to potentially reduce complications and improve patient recovery outcomes.

## References

[1] Bhatia M, Brady M, Shokuhi S, Christmas S, Neoptolemos JP, Slavin J (2000). Inflammatory mediators in acute pancreatitis. J Pathol.

[2] de Pretis N, Amodio A, Frulloni L (2018). Hypertriglyceridemic pancreatitis: epidemiology, pathophysiology and clinical management. United European Gastroenterol J.

[3] Roberts SE, Morrison-Rees S, John A, Williams JG, Brown TH, Samuel DG (2017). The incidence and aetiology of acute pancreatitis across Europe. Pancreatology.

[4] Banks PA, Bollen TL, Dervenis C (2013). Classification of acute pancreatitis - 2012: revision of the Atlanta classification and definitions by international consensus. Gut.

[5] Thoeni RF (2012). The revised Atlanta classification of acute pancreatitis: its importance for the radiologist and its effect on treatment. Radiology.

[6] Aydin H, Tatar IG, Hekimoglu B (2014). The role of diffusion weighted MR imaging in the diagnosis of acute pancreatitis. Int J Emerg Ment Health.

[7] Foster BR, Jensen KK, Bakis G, Shaaban AM, Coakley FV (2016). Revised Atlanta classification for acute pancreatitis: a pictorial essay. Radiographics.

[8] Balthazar EJ, Robinson DL, Megibow AJ, Ranson JH (1990). Acute pancreatitis: value of CT in establishing prognosis. Radiology.

[9] Mortele KJ, Wiesner W, Intriere L (2004). A modified CT severity index for evaluating acute pancreatitis: improved correlation with patient outcome. AJR Am J Roentgenol.

[10] Miller FH, Keppke AL, Dalal K, Ly JN, Kamler VA, Sica GT (2004). MRI of pancreatitis and its complications: part 1, acute pancreatitis. Am J Roentgenol.

[11] Zaheer A, Singh VK, Qureshi RO, Fishman Ek (2013). The revised Atlanta classification for acute pancreatitis: updates in imaging terminology and guidelines. Abdom Imaging.

[12] Shinya S, Sasaki T, Nakagawa Y, Guiquing Z, Yamamoto F, Yamashita Y (2009). The efficacy of diffusion-weighted imaging for the detection and evaluation of acute pancreatitis. Hepatogastroenterology.

[13] Lee NK, Kim S, Kim DU, Seo HI, Kim HS, Jo HJ, Kim TU (2015). Diffusion-weighted magnetic resonance imaging for non-neoplastic conditions in the hepatobiliary and pancreatic regions: pearls and potential pitfalls in imaging interpretation. Abdom Imaging.

[14] Akisik MF, Sandrasegaran K, Jennings SG, Aisen AM, Lin C, Sherman S, Rydberg MP (2009). Diagnosis of chronic pancreatitis by using apparent diffusion coefficient measurements at 3.0-T MR following secretin stimulation. Radiology.

[15] Iranmahboob AK, Kierans AS, Huang C, Ream JM, Rosenkrantz AB (2017). Preliminary investigation of whole-pancreas 3D histogram ADC metrics for predicting progression of acute pancreatitis. Clin Imaging.

